# Supplementary material for: A mathematical model of tumor growth and its response to single irradiation
Source: Theor Biol Med Model. 2016 Feb 27;13:6. doi: 10.1186/s12976-016-0032-7 (PMC4769590; doi:10.1186/s12976-016-0032-7)
Supplement: Additional file 1: — Mathematical details. (DOCX 27 kb) [file 12976_2016_32_MOESM1_ESM.docx]

**Supplement A: Mathematical details**

*Gompertzian solution*

When there is no radiation, i.e., *D* = 0, Equations (2a) and (2c) in the non-dimensionalized form become

$\frac{dy_{1}}{\mathrm{ds}}=2y_{3}y_{1}$ (A.1a)

$\frac{dy_{3}}{\mathrm{ds}}=Ay_{3}$ (A.1b)

Here, s is the time. y_1_ and y_3_ are V_T_ and λ in non-dimension, respectively. By the definition, A < 0. It is straightforward to show that the solution for y_1_ is the Gompertzian function ([Laird, 1964](#_ENREF_26)):

$y_{1}(s)=y_{1}\left( 0 \right)exp\left\{ \frac{2}{A}y_{3}(0)\left[ \exp\left( \mathrm{As} \right)-1 \right] \right\}$ (A.2)

*Estimation of* $\tau_{\mathrm{rad}}$ *from Equation (20)*

It is natural to consider that the number of dividing cells, *N_D_*, decreases exponentially with the time constant of *g(D)* after irradiation at time *t*:

$N_{D}\left( t \right)=N_{D}\left( t_{-} \right)e^{-g(D)t}$ (A.3)

Plugging Equation (A.3) into the left side of Equation (20) and doing the integration, we obtain

$L.H.S.=N_{D}\left( t_{-} \right)\left( 1-e^{-g(D)\tau_{\mathrm{rad}}} \right)$ (A.4)

Meanwhile, the right hand side of Equation (20) is expressed by

$R.H.S.=N_{D}\left( t_{-} \right)\left( 1-e^{-\chi(D)} \right)$ (A.5)

By using the formula for *g(D)* given by Equation (19), hence, it is easy to show

$\tau_{\mathrm{rad}}=3T_{m}$ (A.6)

If *T_m_* is set to ten times *T_cc_* (= 1 day),$\tau_{\mathrm{rad}}$ is 30 days. Note that the value of $\tau_{\mathrm{rad}}$for the clinical cases was set to 8 days as seen in Table 2 (main text). This implies that our model resulted in cumulative cell killing less than the LQ model.
