# Supplementary material for: A mathematical model of tumor growth and its response to single irradiation
Source: Theor Biol Med Model. 2016 Feb 27;13:6. doi: 10.1186/s12976-016-0032-7 (PMC4769590; doi:10.1186/s12976-016-0032-7)
Supplement: Additional file 2: — Comparison of the proposed model with a traditional model. Table S1. Model parameters for rat rhabdomyosarcoma experiment data: the old model(*). Figure S1. Temporal change of the rat rhabdomyosarcoma tumor volume before and after irradiation. The discrete points indicate the experimental data, whereas the solid lines show the tumor volume change produced by the old model. (DOCX 174 kb) [file 12976_2016_32_MOESM2_ESM.docx]

**Supplement B: Comparison of the proposed model with a traditional model**

*Methods and results*

In this supplementary notes, we compared the current model (or “proposed model”) with more traditional model (or “old model”) by applying these models to the data of the rat rhabdomyosarcoma experiment. There are many variations of tumor kinetic models proposed in the literature[[21](#_ENREF_21), [19](#_ENREF_19), [16](#_ENREF_16)]. To simplify the comparison, we used a model in which the Gompetzian growth characteristics is included in the same as the proposed model and the tumor cell death at an the time of instantaneous irradiation is modeled as a sudden change of the volume made of chronogenic cells according to Equations (11a) and (11b). It is noted that the Gompetzian-type growth characteristics is ignored in many old models, which were used for modeling cell proliferation and radiation effects at the same time.[[19](#_ENREF_19)]

For the comparison in this section, we optimized three parameters α, θ, and T_cl_. The final parameter values are given in Table B.1. The initial tumor volume doubling time was set to 1.35 days, which is the same as the value used for the proposed model (see Table 2). The temporal changes of the tumor volume obtained by the old model are plotted in Figure B.1. It must be emphasized that the non-Gompetzian type model with θ=1 cannot replicate the growth curve of the control case.

There are some distinctive differences between the proposed and old models. The α-value of the old model (0.05 ~ 0.09 Gy^-1^) is much smaller than that of the proposed model (0.145 ~ 0.30 Gy^-1^). The data of the α-value were experimentally obtained for two rat rhabdomyosarcoma cell lines and the value is 0.44 Gy^-1^ for the cells containing H-ras oncogene.[[41](#_ENREF_40)] Hence, the proposed model predicts the α-value that is closer to the measurement than the old model. Because of the sudden decrease of the tumor volume at the time of irradiation, the old model shows a sharp change of the tumor volume at that time, whereas the prolonged radiation effect used by the current model can smooth out the volume change after the irradiation.

The MSD values are shown in Table 2 (main text) and Table B.1. Except the control case, for which MSDs are the same, the MSDs of the proposed model is about a half or less than those of the old model. This clearly indicates a better modelling capability by the proposed model in comparison to the old model. The AIC values shown in Table 2 (main text) and Table B.1 also showed the superiority of the new model over the old model even when the number of model parameters and the number of the data points were considered in the model evaluation.

Table B.1: Model parameters for rat rhabdomyosarcoma experiment data: the old model^(*)^

| Parameter\Case | Unit | Control | 1000 | 2000 | 3000 | 4000 |
| --- | --- | --- | --- | --- | --- | --- |
| α | Gy^-1^ |  | *0.08* | *0.07* | *0.05* | *0.04* |
| Dose | Gy |  | 10 | 20 | 30 | 40 |
| T_d_(0) | days | 1.35 | 1.35 | 1.35 | 1.35 | 1.35 |
| θ |  | *0.72* | *0.77* | *0.82* | *0.843* | *0.855* |
| T_cl_ | days |  | 5 | 5 | 5 | 5 |
| N |  | 11 | 11 | 19 | 18 | 14 |
| MSD^(**)^ |  | 0.01883 | 0.02168 | 0.01583 | 0.02722 | 0.05003 |
| AIC^(+)^ |  | -62.07 | -60.52 | -127.61 | -108.90 | -70.88 |

^(*)^ Initial volume = 0.0157cm^3^. The radiation was turned on at the 11^th^ day. The following model parameters were set constant: α/β=10 Gy, cell cycle time T_cc_= 1 day, colony counting time T_m_ = 10 days.

(**) Mean square of differences between the experimental data and the model estimated volumes.

^(+)^ AIC defined by Eq.(23) with *k* = 4. The number of data points, *N*, is given in the table.

Figure B.1: Temporal change of the rat rhabdomyosarcoma tumor volume before and after irradiation. The discrete points indicate the experimental data, whereas the solid lines show the tumor volume change produced by the old model.


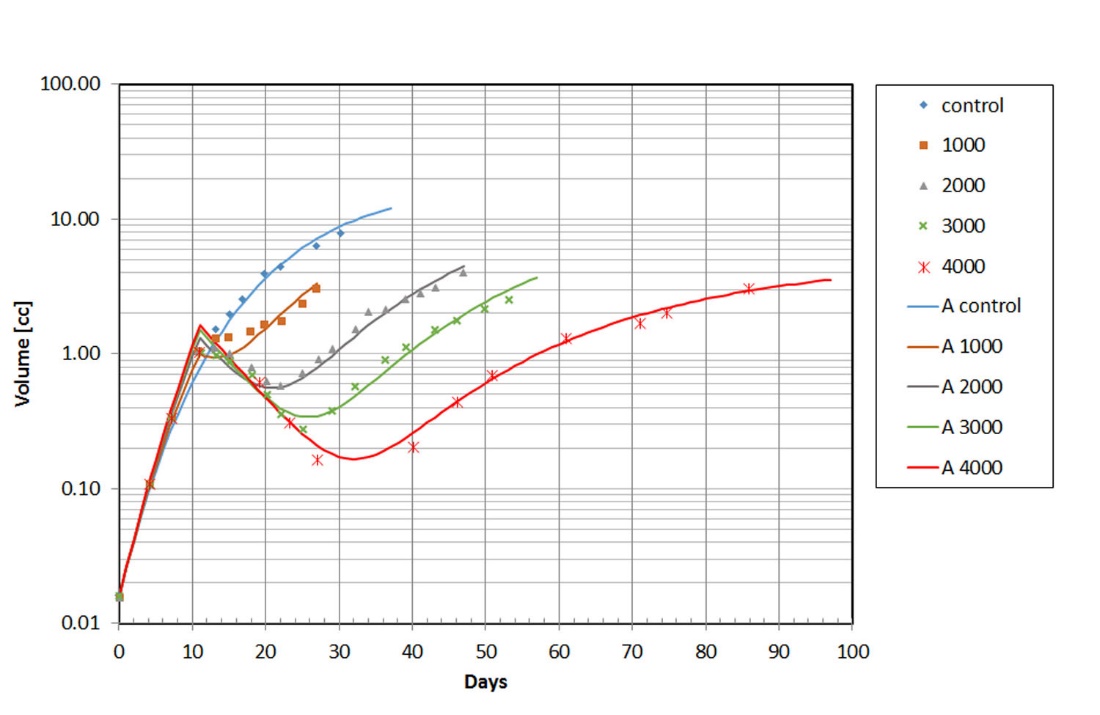


*Discussion and conclusions*

The performance of the new two-component model was compared with an older model by applying those to a rat rhabdomyosarcoma experimental data. The new model proposed in this study included a parameter for the slowing-down process of the tumor growth with increasing tumor size (or the Gompertzian-like tumor growth characteristics) and the prolonging effect of radiation after a pulse of irradiation. We found that both models could reproduce the phenomenological characteristics of the growth and radiation response patterns of tumor volume change for varying radiation dosage. However, first it should be reminded that modeling the slowing down of growth is critical to explain the experimental results. Many models currently used in conjunction with radiation response in the field of radiotherapy do not model the Gompetzian-like growth pattern [[15](#_ENREF_15), [20](#_ENREF_20), [16](#_ENREF_16)]. Secondly, the new model can quantitatively fit the experimental data better than the old model as evidenced by smaller mean square of the differences between modeled volumes and the experimental data. Particularly, the smooth volume change right after a pulse of irradiation can be appropriately modeled only by the new model. Thirdly, one of radiobiological parameters, α, estimated by using the old model turned to be too small in comparison to experimentally determined α-values for this type of cancer cells. In conclusion, we have demonstrated the superiority of the new model over the old model.
